# Anaesthesia for Caesarean Section in a Patient With Familial Amyloid Polyneuropathy Type I

**DOI:** 10.7759/cureus.75033

**Published:** 2024-12-03

**Authors:** Rita R Oliveira, Ana Pereira, Pedro Costa, Ana Ferreira

**Affiliations:** 1 Anesthesiology, Hospital Vila Franca de Xira, Lisboa, PRT

**Keywords:** case report, fap-i, obstetric anaesthesia, peripheral neuropathy, regional anaesthesia

## Abstract

Familial amyloid polyneuropathy type I is a rare neurodegenerative disease characterised by early-onset and progressive peripheral sensorimotor and autonomic polyneuropathy. A pregnant woman with familial amyloid polyneuropathy type I presented with mild gastrointestinal, cardiac involvement and peripheral neuropathy, aggravated during the pregnancy. The caesarean delivery was performed under uneventful epidural anaesthesia. Anaesthesia of a pregnant woman with this condition can be challenging due to its multisystemic involvement and the possibility of further neurologic injury. This is one of the few cases of anaesthesia for caesarean section described in familial amyloid polyneuropathy type I patients. Given the possibility of similar cases, it is essential to disclose the anaesthetic management.

## Introduction

Familial amyloid polyneuropathy type I (FAP-I) is a rare disorder caused by mutations in the transthyretin (TTR) gene that leads to misfolding of TTR proteins, which aggregate as amyloid deposits in multiple tissues. The largest clusters can be found in Portugal, Brazil, and Japan. It affects both genders equally, affecting mainly young adults [1].

FAP-I is an autosomal dominant inherited condition, a rapidly progressive and early-onset form of peripheral sensorimotor and autonomic neuropathy. Airway amyloid infiltration, ocular deposition, nephropathy, and gastrointestinal and cardiac impairment can also occur [2]. The available disease-modifying treatments include liver transplants and tafamidis. If untreated, the disease can progress to death within 10 years of onset [3].

The descriptions in the literature of anaesthesia in FAP-I patients are mostly in the context of liver transplants. There is some information regarding a neuraxial approach, none of which is in the context of an elective caesarean section. The potential risks of both anaesthetic techniques on these patients, associated with the mother's desire to be awake during the baby's birth, impose a significant challenge in anaesthetic management for these patients.

## Case presentation

A 36-year-old woman with FAP-I was scheduled for elective caesarean delivery at 39 weeks gestation (due to a previous caesarean section). The onset of symptoms was one year before admission, with postprandial fullness. One month later, she developed neuropathic pain with thermal hypoesthesia and paraesthesia in both feet (neuropathy impairment score (NIS) of 5), followed by self-limited episodes of diarrhoea. A salivary gland biopsy revealed amyloid deposits, electromyographic evaluation showed signs of abnormal sural nerve conduction, and the patient also had a previously known first-degree atrioventricular block. There were no signs of motor or kidney impairment. The patient had a good functional capacity and did not have signs of cardiac autonomic dysfunction. The patient had a stage I polyneuropathy disability score (PDS) and was treated with 20 mg tafamidis once a day and pregabalin 75 mg + 150 mg, with moderate symptom control.

The pregnancy was unplanned. Treatment with tafamidis and pregabalin was terminated at the third week of gestation and was replaced with paracetamol thrice daily. From the second trimester onwards, the patient reported worsening neuropathic symptoms with increased paraesthesia and pain evoked by light touch until the lower third of the legs, with an NIS of 6.

At admission, the neurological symptoms were barely controlled. The laboratory results were unremarkable. The airway assessment showed no signs of potential difficulty. The benefits and risks were explained, and the patient opted for a regional technique.

On arrival in the delivery room, blood pressure was 128/87 mmHg, heart rate was 83 beats per minute (bpm-1), and peripheral O_2_ saturation (SpO_2_) was 100%. Standard ASA (American Society of Anesthesiologists) monitoring was used. Temperature was monitored, and active external warming was implemented.

An epidural block was performed at the L3-L4 intervertebral level. The technique was linear and atraumatic, without reference to symptoms. Ropivacaine 0.75% 10 ml plus sufentanil 10 µg (total of 12 ml) were administered via the epidural catheter. We performed a coloading during the procedure and administered a total of 500 ml of balanced crystalloid solution during surgery. The patient was positioned in a left lateral tilt, and foetal monitoring was assured until the epidural block was complete. After 25 minutes, there was a sensory block until T4, and the surgery began. Four minutes later, the baby was delivered uneventfully.

The patient was stable throughout the procedure (Table 1) with no need for vasopressors. Oxytocin was administered according to hospital protocol (perfusion of 20 U). At the end of the surgery, paracetamol 1000 mg intravenous (IV), ketorolac 30 mg IV, and morphine 1.5 mg epidural were administered. Nausea and vomiting prophylaxis were achieved with dexamethasone 4 mg and ondansetron 4 mg.

**Table 1:** Vital signs during the caesarean section DBP: Diastolic blood pressure; DR: Delivery room; HR: Heart rate; MAP: Mean arterial pressure; SpO_2_: Peripheral oxygen saturation; SBP: Systolic blood pressure; PACU: Post-anaesthesia care unit

Timing	SpO_2_ (%)	HR (bpm^-1^)	SBP (mmHg)	DBP (mmHg)	MAP (mmHg)
Entrance on the DR	100	83	148	87	114
Beginning of anaesthesia	100	89	128	87	105
Epidural bolus	100	95	119	79	95
10 minutes after epidural bolus	100	109	129	82	94
20 minutes after epidural bolus	100	112	117	74	88
Beginning of surgery	99	115	128	85	102
Delivery	99	87	124	76	94
End of surgery	99	97	109	53	74
End of anaesthesia	99	93	112	68	82
PACU	99	82	117	75	90

After approximately three hours and the fulfilment of the Aldrete criteria, upon post-anaesthetic care unit discharge, the numeric pain rating scale (NPRS) at the incision site was zero at rest and 1-2 with movement. With the paediatrician's consent, pregabalin 75 mg was resumed on that night with a progressive increase. On postoperative day one, the pain at the incision site was mild.

There were no signs of neurological deterioration related to peripheral neuropathy prior to discharge, which occurred on day three after surgery.

Two months post-caesarean, the patient had a neurology follow-up, in which she disclosed similar symptoms to the ones on the day of surgery without further exacerbation. She resumed tafamidis and increased pregabalin to 150+150 mg due to the symptom exacerbation since the second trimester of the pregnancy. When asked about the childbirth experience, she was satisfied with the provided care, and her wishes and expectations were fulfilled.

## Discussion

FAP-I is a progressive, uncommon, and neurodegenerative disease with multisystemic involvement and broad clinical presentation [4]. The evidence regarding its anaesthetic management is scarce, especially concerning regional techniques and obstetrics [5].

Pre-anaesthetic assessment is essential in polyneuropathies such as FAP-I due to multisystemic involvement. Past medical history should be taken on admission, and any anaesthetic complications should be recorded. A physical examination and neurologic evaluation should also be conducted [6].

It is crucial to evaluate the degree of sensory, motor, and autonomic impairment in order to plan the ideal anaesthesia technique for monitoring and to anticipate possible complications and postoperative care. Several scoring systems are described, which include PDS and the FAP staging system for disease staging and NIS for standard, quantitative, reproducible neurological impairment evaluation [7]. The patient was considered to be stage I in PDS; she had sensory disturbances without an ambulatory deficiency.

Cardiac involvement is the most common extra-neurological manifestation observed in 80% of the patients. Standard clinical features in cardiac amyloidosis include arrhythmias, syncope due to conduction abnormalities, and heart failure caused by restrictive cardiomyopathy. The patient already had an asymptomatic first-degree atrioventricular block. Therefore, considering the high prevalence of cardiac impairment, a recent cardiac evaluation, including an electrocardiogram, echocardiography, and brain natriuretic peptide/troponin measurement, is advisable [8].

Preoperative knowledge of autonomic neuropathy is crucial with these patients. Haemodynamic fluctuations are one of the chief symptoms and should be assessed preoperatively. The response to cardiovascular vasopressor drugs may be exaggerated, while the response to vasoactive agents is unpredictable. Gastrointestinal autonomic impairment is also common, causing a higher risk of gastroparesis and aspiration, particularly worrying in pregnant patients. Euthermia should be maintained due to inadequate thermal regulation. Complete blood count, electrolyte balance, and renal function should also be reviewed in case of possible ongoing nephropathy, as we did [8-10].

A neuraxial block is an anaesthetic technique usually performed for caesarean deliveries due to the following reasons: no need to approach the airway, newborns do not get exposed to the depressant effect of anaesthetic agents, lower risk of lung aspiration, and early establishment of a bond between the mother and newborn [11].

An epidural block was chosen because of its haemodynamic stability, better analgesia, attenuation of the stress response, and avoidance of polypharmacy [7]. Maternal preferences should also be considered, promoting a successful individual birth experience.

Regardless of the underlying aetiology, the presence of chronic neural compromise derangements may place patients at increased risk of further neurologic injury through the 'double crush phenomenon'. Given the lack of clinical evidence, definitive recommendations cannot be made regarding the safety and use of regional anaesthesia in patients with pre-existing inherited peripheral neuropathies. Isolated case reports suggest that regional techniques may be used without worsening a patient's stable neurological condition. As a general rule, a reduced concentration or dose of a local anaesthetic should be used [8].

In this case, the patient reported progression of neuropathic symptoms since the second trimester of pregnancy. Although we cannot completely rule out interference of the anaesthetic technique, this seems unlikely. It is safer to assume that it is related to the suspension of treatment with tafamidis.

In this particular population, it is also essential to evaluate the risk of bleeding. FAP-I can be associated with increased risk, even with normal coagulation times, caused by platelet dysfunction, fibrinolysis impairment, and coagulation factor deficit [6]. Nevertheless, there are no reported cases of epidural haematomas in this population. When choosing between epidural or spinal techniques, an epidural has the advantage of allowing a slower block installation as well as a less intense sympathetic block.

For postoperative management, it is important to evaluate neurological status after the block wears off and consider the postoperative management of pain and neuropathic complaints. Multidisciplinary involvement throughout the perioperative period is essential to optimising treatment.

## Conclusions

To our knowledge, this is one of the few cases of anaesthesia for caesarean section described in FAP-I patients. Given the possibility of similar cases, it is essential to disclose the anaesthetic management in this patient and give a perspective on the experience, as it was difficult to identify the ideal approach.

Although there are no specific reports regarding this population, considering other peripheral neuropathies, it seems safe to assume that a neuraxial technique can be used in these patients, as in the general population.
